# P53-Induced Autophagy Degradation of NKX3-2 Improves Ovarian Cancer Prognosis

**DOI:** 10.3390/cells14110765

**Published:** 2025-05-22

**Authors:** Alessandra Ferraresi, Ian Ghezzi, Amreen Salwa, Chiara Lualdi, Danny N. Dhanasekaran, Ciro Isidoro

**Affiliations:** 1 Laboratory of Molecular Pathology, Department of Health Sciences, Università del Piemonte Orientale, Via Solaroli 17, 28100 Novara, Italy; 20020210@studenti.uniupo.it (I.G.); salwa.amreen@uniupo.it (A.S.); chiara.lualdi@uniupo.it (C.L.); 2 Stephenson Cancer Center, The University of Oklahoma Health Sciences Center, Oklahoma City, OK 73104, USA; danny-dhanasekaran@ouhsc.edu

**Keywords:** BAPX1, p53, autophagy, lysosome, apoptosis, cancer cell metabolism, TCGA, prognosis, personalized medicine, ovarian cancer

## Abstract

NKX3-2, a transcriptional repressor factor belonging to the NK family of homeobox-containing proteins, has been widely studied for its role in promoting chondrogenic differentiation and homeostasis. NKX3-2 is upregulated in chemoresistant ovarian tumors and metastatic gastric cancer cells; however, its prognostic role and mechanistic involvement in cancer cell biology remain to be elucidated. By interrogating the TCGA database, we found that cancer patients with high *NKX3*-*2* expression had a shorter overall survival rate than patients with low expression. In ovarian cancer patients, NKX3-2 negatively correlates with P53. Given the prominent role of the latter oncosuppressor in controlling DNA repair and cell death, here we investigate the molecular mechanism involved in this negative correlation in several ovarian cancer cell lines expressing different levels of the two proteins. We found that the high expression of endogenous or ectopic P53 reduced NKX3-2 protein expression, while its knockdown increased it. In contrast, the genetic manipulation of NKX3-2 expression did not affect P53 expression. Mechanistically, P53-mediated downregulation of NKX3-2 does not entail transcriptional activity or proteasomal clearance but occurs via P53–NKX3-2 protein–protein interaction, which in turn results in P53-induced NKX3-2 degradation via the autophagy–lysosome pathway. Remarkably, patients bearing a tumor characterized by low *NKX3-2* and high *MAP1LC3B* expression (indicative of active autophagy) display a better prognosis. Taken together, our data indicate that NKX3-2 represents a negative prognostic factor under P53 control in ovarian cancer. From a translational point of view, identifying this novel mechanism may represent a new molecular signature capable of predicting the clinical outcome of patients, a crucial aspect of developing personalized therapeutic approaches.

## 1. Introduction

Ovarian cancer continues to be a deadly malignancy, accounting for the highest mortality rate compared to other gynecological tumors [1]. The high heterogenicity of ovarian tumors represents a serious challenge toward a ‘one size fits all’ therapy. A precision medicine approach is desirable in the development of more effective therapeutic strategies for ovarian cancer, and this requires the identification of novel reliable prognostic indicators able to predict the therapy response and long-term outcome [2].

NKX3-2 (also known as Bapx1) is a transcriptional repressor widely studied for its role in promoting chondrocyte differentiation and homeostasis [3,4]. NKX3-2 has not been widely investigated in relation to tumor development and progression, with a few studies primarily describing its ability to facilitate gastric cancer metastasis [5] and the dysregulation of immune cell differentiation programs in B-cell lymphomas and T-cell acute lymphoblastic leukemia [6,7]. In ovarian serous carcinoma, NKX3-2 overexpression was associated with distant metastasis, especially in chemo-resistant tumors compared to chemo-sensitive ones [8]. Recently, we have reported that NKX3-2 promotes ovarian cancer cell migration by downregulating autophagy through the modulation of lysosome transport [9]. Overall, these findings indicate that NKX3-2 could act as an oncogenic protein, thus prompting us to investigate its potential as a prognostic marker.

We found that high *NKX3*-*2* expression predicts unfavorable clinical outcomes in different solid tumors. Further, TCGA interrogation and in vitro findings indicated that NKX3-2 negatively correlates with the oncosuppressor P53. Remarkably, the transcriptomic analysis revealed that ovarian cancer patients with low *NKX3-2*/high *TP53* expression display a marked downregulation of a wide range of genes related to oncogenic hallmarks (e.g., apoptosis evasion, cell migration, macromolecule biosynthetic processes, etc.) in parallel to an upregulation of several transcripts involved in DNA damage checkpoints, mitochondrial respiration, and proteolysis. Mechanistically, here we show for the first time that P53 interacts with NKX3-2, which in turn results in P53-induced autophagy degradation of NKX3-2. Accordingly, P53 ectopic overexpression suppresses, while P53 silencing rescues NKX3-2 protein levels. Additionally, we experimentally proved that NKX3-2 binds to the autophagosomal protein LC3 in P53-proficient cells. Notably, we found that a low *NKX3-2*/high *MAP1LC3B* signature predicts a better prognosis for ovarian cancer patients. Taken together, our data reveal that the oncosuppressor P53 counteracts NKX3-2 expression in ovarian cancer cells and supports the prognostic value of NKX3-2 for the stratification of cancer patients in a view of precision medicine.

## 2. Materials and Methods

### 2.1. Cell Cultures

Three human ovarian cancer cell lines differing in *TP53* status were employed in the study: SKOV3, OVCAR3, and OAW42. SKOV3 and OVCAR3 cells (both purchased from ATCC, Manassas, VA, USA) were grown in RPMI 1640 media (cod. R8758; Sigma Aldrich, St. Louis, MO, USA) enriched with 10% FBS (cod. ECS0180L; Euroclone, Milan, Italy), 1% penicillin/streptomycin solution (cod. P0781; Sigma Aldrich), and 1% glutamine (cod. G7513; Sigma-Aldrich). OAW42 cells (purchased from EACC, Porton Down, Salisbury, UK) were grown in MEM media (cod. M2279; Sigma-Aldrich) enriched with 10% FBS, 1% penicillin/streptomycin, 1% non-essential amino acids (cod. M7145; Sigma-Aldrich), and 1% glutamine.

### 2.2. Reagents and Antibodies

Chloroquine (ClQ, cod. C6628; Sigma Aldrich), a lysosome alkalinizing drug that impairs autophagosome-lysosome fusion, was used at 30 μM final concentration. MG132 (cod. M7449; Sigma Aldrich), a proteasome inhibitor that reversibly inhibits the enzymatic activity of the proteasome, was used at 10 μM final concentration. Nutlin-3a (cod. SML0580; Sigma Aldrich), an inhibitor of MDM2, was used at 5 µM final concentration.

The following primary antibodies were used: mouse anti-p53 (1:200, cod. sc-126; Santa Cruz Biotechnologies, Santa Cruz, CA, USA), goat anti-p53 (1:50, cod. sc-6243; Santa Cruz Biotechnologies), rabbit anti-NKX3-2 (1:500, cod. PA5-21108; Invitrogen, Waltham, MA, USA), rabbit anti-LC3 (1:1000, cod. L7543; Sigma Aldrich), mouse anti-LAMP1 (1:1000, cod. 555798; BD Biosciences, Franklin Lakes, NJ, USA), rabbit anti-GAPDH (1:1000, cod. G9545; Sigma Aldrich), mouse β-Actin (1:2000, cod. A5541; Sigma Aldrich), and mouse β-Tubulin (1:1000, cod. T5201; Sigma Aldrich).

The following secondary antibodies were employed for Western blotting: HRP-conjugated goat anti-mouse (cod. 170-6516) and goat anti-rabbit (cod.170-6515) (both diluted 1:10,000, BioRad, Hercules, CA, USA). The following secondary antibodies were employed for immunofluorescence: AlexaFluor594-conjugated donkey anti-goat IgG antibody (cod. A11058), AlexaFluor488-conjugated goat-anti-rabbit IgG antibody (cod. A32731), and AlexaFluor555-conjugated goat-anti-mouse IgG antibody (cod. A32727) (diluted 1:1000, Thermo Fisher Scientific, Waltham, MA, USA).

### 2.3. Transfection

Cells were seeded on coverslips or Petri dishes (as indicated in figure legends) and allowed to adhere and reach the appropriate confluence (about 24–36 h) before proceeding with the transfection.

Gene silencing was achieved by transfecting the cells with 150 pmol siRNA using Lipofectamine 3000 Reagent (cod. L3000-015, Life Technologies, Paisley, UK). After 72 h from the transfection, samples were analyzed by immunofluorescence, immunoprecipitation, or Western blotting. siRNAs sequences were as follows: 5′-CCAAGAAGGUGGCCGUAAAUU-3′ for siNKX3-2, 5′-AAGAAACCAACUGGAUGGAGAAUAUUUC-3′ for siP53, and 5′-AGGUAGUGUAAUCGCCUUGTT-3′ for siRNA scramble.

P53 overexpression was achieved by transient transfection with 2 µg of pcDNA 3.1-Zeo(-)-P53 using the Lipofectamine 3000 Reagent, following the manufacturer’s instructions. After 72 h from the transfection, samples were analyzed by Western blotting, immunoprecipitation, or immunofluorescence.

### 2.4. Nuclear/Cytoplasmic Extraction

Cells were plated in Petri dishes, transfected, and post-transfection cultured as detailed in the figure legends. Cells were collected by trypsinization and centrifuged at 500× *g* for 5 min, and the pellets were washed with PBS and then subjected to differential extraction of the nuclear and cytoplasmic fractions using NE-PER kit (cod. 78833; Thermo Fisher Scientific) following the manufacturer’s instructions. Nuclear and cytoplasmic proteins were quantified by BCA protein assay, and the proteins of interest were detected by Western blotting.

### 2.5. Western Blotting

Protein homogenates were prepared following the standard procedure described in [9]. Samples were separated by SDS-PAGE and then blotted on a PVDF membrane. The saturated filters were incubated overnight at 4 °C with a specific primary antibody solution. The following day, the membranes were washed and incubated with a solution of secondary HRP-conjugated antibody for 1 h at room temperature. The detection of the bands was achieved using Enhanced Chemiluminescence reagents (cod. NEL105001EA; Perkin Elmer, Waltham, MA, USA), and the results were imaged at the VersaDOC Imaging System (BioRad). The normalization of data was performed by re-probing the membranes for β-Actin, β-Tubulin, or GAPDH. Densitometric analysis was performed using Quantity One software (v.4.5). The densitometric data are reported in arbitrary units.

### 2.6. Immunofluorescence

Cells were seeded on sterile coverslips and treated or transfected as detailed in the figure legends. The coverslips were fixed in methanol, permeabilized, and stained overnight at 4 °C with specific primary antibodies. The subsequent day, the coverslips were washed and stained with a solution containing dye-conjugated secondary antibodies and DAPI for 1 h at room temperature. After the mounting with the SlowFade reagent (cod. S36936; Invitrogen), the coverslips were acquired using a fluorescence microscope. The integrated fluorescence values (Int DEN) were determined using the ImageJ software (v. 1.48; NIH). For co-localization assays, the graphs display the quantification of the combined fluorescence intensity derived from the merging of the fluorescent channels (representing the close proximity between NKX3-2/P53, NKX3-2/P53/LC3, and NKX3-2/P53/LAMP1).

### 2.7. Image Acquisition and Analysis

Fluorescence images were captured using a fluorescence microscope (Leica DMI6000). For every experimental condition, five to ten randomly selected microscopic fields were acquired by two independent researchers unaware of the treatment. The images displayed in the panels are representative of three independent replicates. For the quantitative analysis of fluorescence intensity, at least 100 to 150 cells were considered.

### 2.8. Immunoprecipitation

Cells were plated in Petri dishes and cultured as described in the figure legends. Before harvesting, cells were incubated for 15 min with 1 mM DTSP (cod. D3669, Sigma Aldrich), a chemical crosslinker that stabilizes even weak interactions. Cells were harvested in lysis buffer, and the sample concentration was estimated by BCA assay. For each experimental condition, 500 μg of protein lysate was incubated with the specific primary antibody (5 μg), and the immunocomplexes were captured with 50 μL of Sepharose G beads (cod. 17061801; Cytiva, Uppsala, Sweden). The immunocomplexes were pulled down by centrifugation and eluted with Laemmli buffer. Samples were loaded on an SDS-PAGE and immunoblotted with specific antibodies to assess the P53/NKX3-2 and NKX3-2/LC3 interactions.

### 2.9. RNA Isolation and Quantitative PCR

Cells were plated in Petri dishes and cultured as detailed in the figure legends. The total RNA was isolated using TRIzol reagent (cod. T9424, Sigma-Aldrich). The mRNA was reverse transcribed into cDNA using the RevertAid First Strand cDNA Synthesis Kit (cod. K1622, Life Technologies, Waltham, MA, USA) following the manufacturer’s instructions. PCR amplification of the target markers was performed with recombinant Taq DNA polymerase (cod. 10342-020, Life Technologies). The PCR products were analyzed by agarose gel electrophoresis. The PCR primers used are the following: p53 (forward 5′-ACACTTTGCGTTCGGGCTGGG-3′; reverse 5′-TCCAGGGTGTGGGATGGGGTG-3′), NKX3-2 (forward 5′-TTACCCGTACTACTGCCTCC-3′; reverse 5′-CTCCTTACATTCAGCACCCG-3′), and β-Actin (forward 5′-GATCAAGATCATTGCTCCTCCTGAGCGCA-3′; reverse 5′-GTCTCAAGTCAGTGTACAGGTAAGCCCT-3′).

### 2.10. Statistical Analysis

The data in the histograms are expressed as the average ± S.D. GraphPad Prism 5.0 was used to perform the statistical analysis. For comparison between three or more experimental groups, we chose Bonferroni’s test after one-way ANOVA analysis (unpaired, two-tailed). For comparison between the two experimental groups, we selected *t*-test analysis (unpaired, two-tailed). Significance was considered as follows: **** *p* < 0.0001; *** *p* < 0.001; ** *p* < 0.01; * *p* < 0.05; not significant (ns) *p* > 0.05.

### 2.11. TCGA Database Interrogation

The data of patients diagnosed with different tumors were retrieved from The Cancer Genome Atlas (TCGA) (https://www.cbioportal.org, last accessed on 31 January 2025). TCGA gene expression profiles and clinical data (e.g., overall survival months) were downloaded from cBioportal.org. The analysis was conducted on different cancer-specific datasets (ovarian cystadenocarcinoma, brain lower-grade glioma, colorectal adenocarcinoma, kidney renal clear-cell carcinoma, liver hepatocellular carcinoma, and breast-invasive carcinoma). Patients were divided into low or high groups based on the median mRNA expression level. Statistical analyses were conducted using R (v.3.6.1; The R Foundation for Statistical Computing, Vienna, Austria) and SAS software (v.9.4; SAS Institute Inc., Cary, NC, USA).

The survival curves of differential expression groups were represented in the form of Kaplan–Meier plots, and the Cox regression model was used to analyze the comparisons. To analyze the statistical significance of survival curves, a log-rank test was employed. A *p*-value < 0.05 was considered significant.

Scatter plots were obtained to represent the correlations between the expression of *TP53* and *NKX3-2* in the ovarian cancer patients’ cohort. Pearson’s and Spearman’s correlation coefficients (r) and the relative *p*-values were calculated to establish the regression model.

RNA-seq data were retrieved from the TCGA repository (Ovarian cystadenocarcinoma dataset, Nature 2011). The identification of the differentially expressed genes (DEGs) was obtained using TBtool (https://github.com/CJ-Chen/TBtools/, accessed on 15 February 2025). An enrichment analysis of DEGs was performed using the DAVID bioinformatics functional annotation tool (https://david.ncifcrf.gov/summary.jsp, accessed on 20 February 2025), which provides Gene Ontology (GO) biological processes and Kyoto Encyclopedia of Genes and Genomes (KEGG) pathways. The bar histograms represent the number of transcripts belonging to each positively and negatively correlated biological process obtained from DAVID analysis. The heatmaps reporting the transcriptomic data were created using MeV4 software (https://webmev.tm4.org/, accessed on 25 March 2025).

## 3. Results

### 3.1. NKX3-2 Negatively Correlates with TP53 Expression and Predicts Unfavorable Clinical Outcomes in Ovarian Cancer

To determine whether NKX3-2 expression could have clinical relevance in cancer, we first focused on the prognostic value of NKX3-2 among various tumors interrogating the TCGA database. Six different datasets representing different solid tumors (including ovarian cancer, brain glioma, colorectal adenocarcinoma, kidney carcinoma, liver cancer, and breast-invasive carcinoma) were considered, and all of them showed a similar trend, indicating that NKX3-2 is a negative prognostic marker. In particular, patients who expressed high levels of *NKX3-2* mRNA showed shorter overall survival than those who expressed low levels (Supplementary Figure S1).

**Figure 1:**
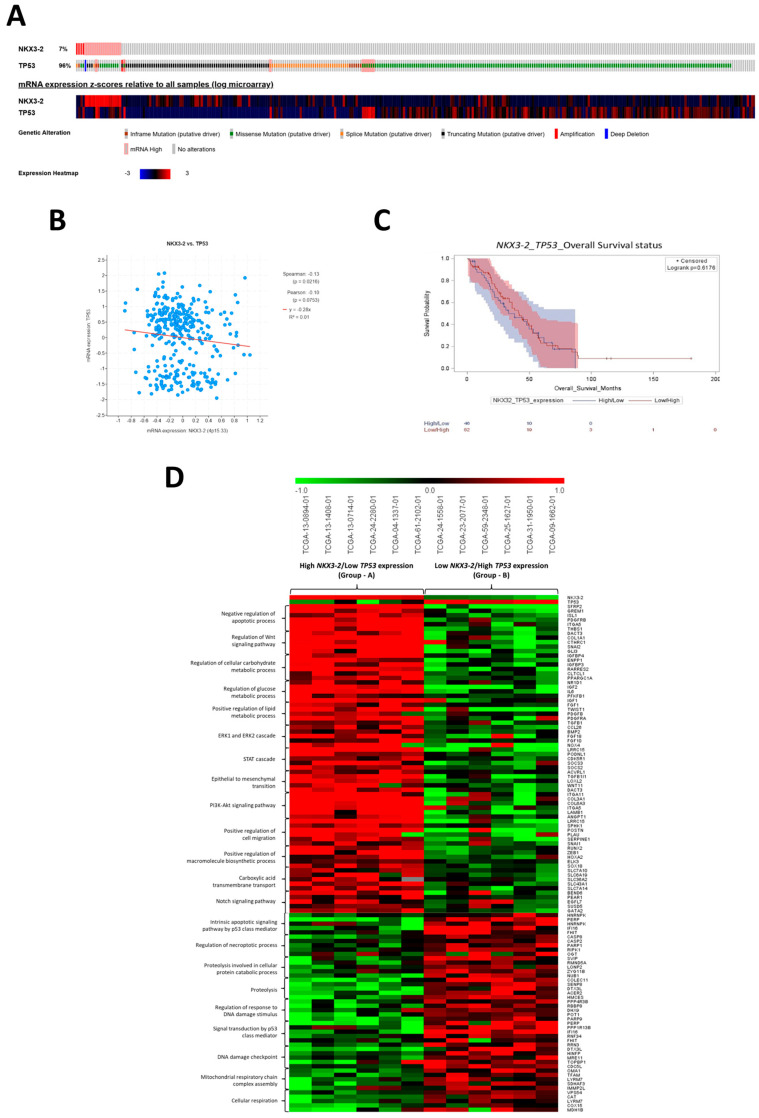
*NKX3-2* negatively correlates with *TP53* expression, and this is associated with poor clinical outcomes. (**A**) The oncoprint shows the genetic alterations and mRNA expression levels of *NKX3-2* and *TP53* in 316 ovarian cancer patients (Ovarian Serous Cystadenocarcinoma dataset, Consortium TCGA, Nature 2011). The heatmap reports upregulated and downregulated genes in red and blue, respectively. (**B**) The scatter plot displays the inverse correlation of *NKX3-2* and *TP53* mRNA levels. (**C**) The Kaplan–Meier curve depicts overall survival based on *NKX3-2*/*TP53* differential expression levels. (**D**) Comparison of differentially expressed genes in two groups of patients stratified based on *NKX3-2* and *TP53* expression. Patients were stratified in Group A (high *NKX3-2*/low *TP53* expression) and Group B (low *NKX3-2*/high *TP53* expression). The heatmap reports the top differentially expressed genes belonging to each biological process related to cell metabolism, cell motility, apoptosis, DNA repair mechanisms, and oncogenic signaling pathways.

In a previous transcriptomic analysis conducted in ovarian cancer, we reported that *NKX3-2* positively correlates with genes involved in resistance to apoptosis and negatively correlates with transcripts belonging to the P53-related signaling pathway [9]. The latter finding prompted us to investigate the possible link between NKX3-2 and P53 more in-depth, given the prominent role of this oncosuppressor in controlling DNA repair and cell death, ultimately influencing the patients’ response to chemotherapy. The upper part of the oncoprint shows that only 7% of ovarian cancer patients display genetic alterations in *NKX3-2*, while 96% of the cohort was altered for the *TP53* gene (Figure 1A). We performed a correlation analysis by monitoring the mRNA expression of these two genes. Remarkably, the heatmap in the bottom part of the oncoprint and the scatter plot collectively show that *NKX3-2* and *TP53* were inversely correlated (Figure 1A,B). More relevantly, we observed that patients bearing tumors with high *NKX3-2* expression together with low *TP53* levels displayed an unfavorable clinical outcome associated with shorter overall survival. In contrast, those presenting a tumor with low *NKX3-2* along with high *TP53* expression exhibit a better prognosis (Figure 1C). Moving forward, we addressed how the NKX3-2/P53 axis impacts the clinical outcome of ovarian cancer patients. In the subsequent analysis, ovarian cancer patients were divided into two groups based on the opposite expression of *NKX3-2* and *TP53*. We selected six patients for each group as follows: (i) Group A included patients with high *NKX3-2*/low *TP53* expression, and (ii) Group B included those with low *NKX3-2*/high *TP53* expression. The heatmap reported in Figure 1D depicts the top significant differentially expressed genes (DEGs) selected for the main biological processes reported in Supplementary Figure S2. The transcriptomic analysis emphasizes that patients in Group A (showing high *NKX3-2*/low *TP53* expression and poor prognosis) were characterized by the upregulation of a range of transcripts involved in negative regulation of apoptosis (*SFRP2*, *GREM1*, *ISL1*, *PDGFRB*, *ITGA5*, and *THBS1*), cell motility (*LRRC15*, *SPHK1*, *POSTN*, *PLAU*, *SERPINE1*, *SNAI1*, and *TWIST1*), carbohydrates metabolism (*IGFBP3*, *IGFBP4*, *RARRES2*, *CTLCL1*, *PPARGC1A*, *ENPP1*, *NR1D1*, *IGF2*, *PFKFB1*, and *IGF1*), lipid metabolism (*FGF1*, *PDGFB*, *PDGFRA*, and *TGFB1*), and macromolecules biosynthesis (*RUNX2*, *ZEB1*, *HOXA2*, *ELK3*, and *SOX1B*). In contrast, patients of Group B (showing low *NKX3-2*/high *TP53* expression and better prognosis) showed a marked downregulation of genes involved in the oncogenic pathways listed above, along with the upregulation of a wide range of transcripts involved in P53-mediated intrinsic apoptosis (*HNRNPK*, *PERP*, *IFI16*, and *FHIT*), proteolysis (*SVIP*, *RMND5A*, *LONP2*, *ZYG11B*, *NUB1*, *COLEC11*, *SENP8*, *DTX3L*, *ACER2*, and *HMCES*), DNA damage checkpoints (*HINFP*, *MRE11*, *TOPBP1*, and *CDC5L*), and oxidative phosphorylation pathway (*VSP54*, *CAT*, *LYRM7*, *COX15*, and *MDH1B*).

Taken together, these data reinforce the view that NKX3-2 may represent an oncogenic factor for ovarian cancer patients.

### 3.2. P53 Interacts with NKX3-2 and Negatively Regulates Its Expression

**Figure 2:**
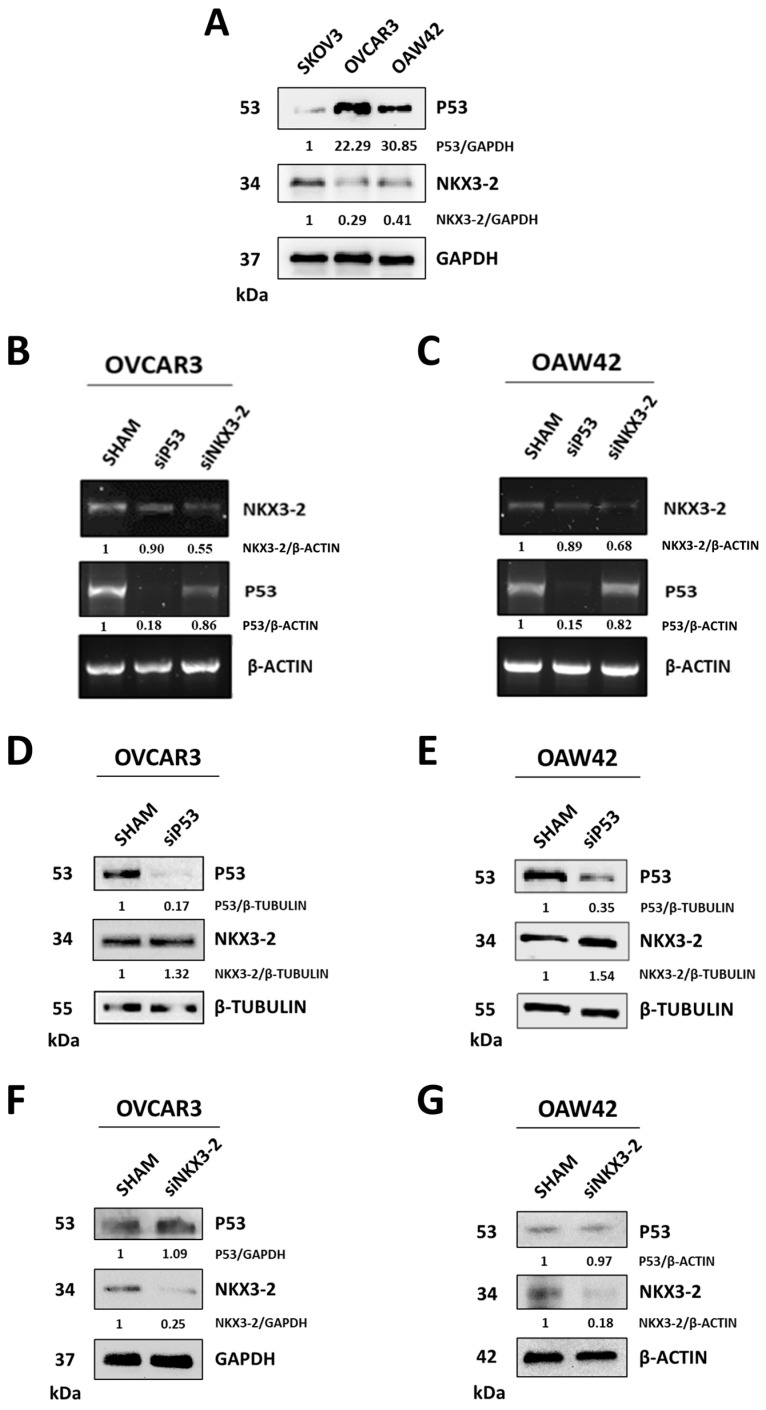
P53 negatively modulates NKX3-2 protein but not vice versa. (**A**) Three cell lines (SKOV3, OVCAR3, and OAW42), representing the heterogenicity of *TP53* (null, mutated, and wild type, respectively), were analyzed for the expression of NKX3-2 by Western blotting. (**B**,**C**) OVCAR3 and OAW42 cells were silenced for P53 or NKX3-2. Agarose gel electrophoresis was used to visualize the PCR products for *P53* and *NKX3-2*. Normalization was performed with *β-ACTIN*. (**D**,**E**) OVCAR3 and OAW42 cells were silenced for P53, and NKX3-2 protein expression was monitored by Western blotting. Normalization was performed with β-Tubulin. (**F**,**G**) OVCAR3 and OAW42 cells were silenced for NKX3-2, and P53 protein expression was monitored by Western blotting. Normalization was performed GAPDH or β-Actin. All densitometric analyses for Western blotting and agarose gel electrophoresis of PCR products are included.

Next, we validated this correlation by assessing NKX3-2 and P53 protein expression in three ovarian cancer cell lines with different genetic backgrounds. In agreement with the in silico data, we observed that OVCAR3 and OAW42 cells (expressing *TP53* mutated and *TP53* wild type, respectively) display a low expression of NKX3-2, while SKOV3 cells (*TP53* null) exhibit the highest levels of NKX3-2 (Figure 2A). Thus, we investigated the mechanism through which P53 can modulate NKX3-2 expression or vice versa. To address whether this negative correlation is transcriptionally governed, we monitored *NKX3-2* and *P53* mRNA levels in genetically manipulated OVCAR3 and OAW42 cells to silence P53 or NKX3-2. The silencing of either NKX3-2 or P53 did not alter *P53* and *NKX3-2* mRNA expression reciprocally (Figure 2B,C), indicating that the molecular mechanism does not entail transcriptional regulation. Consistently, the cytoplasmic/nuclear ratios of NKX3-2 expression do not exhibit any significant change upon P53 overexpression (in SKOV3) or P53 silencing (in OVCAR3 and OAW42), respectively (Supplementary Figure S3). Next, we monitored the expression of these two proteins in the same experimental conditions described above. We observed that the knockdown of P53 in both OVCAR3 and OAW42 cell lines leads to an increase in NKX3-2 expression (Figure 2D,E). As a further confirmation, OAW42 cells (*TP53* wild type) incubated with Nutlin-3, a potent inhibitor of MDM2 that stabilizes P53, displayed about 50% decrease in NKX3-2 protein expression compared to that of the control (Supplementary Figure S4). Notably, we found that NKX3-2 knockdown does not significantly change P53 levels in both cell lines (Figure 2F,G), indicating that the regulation of P53 on NKX3-2 protein is monodirectional.

**Figure 3:**
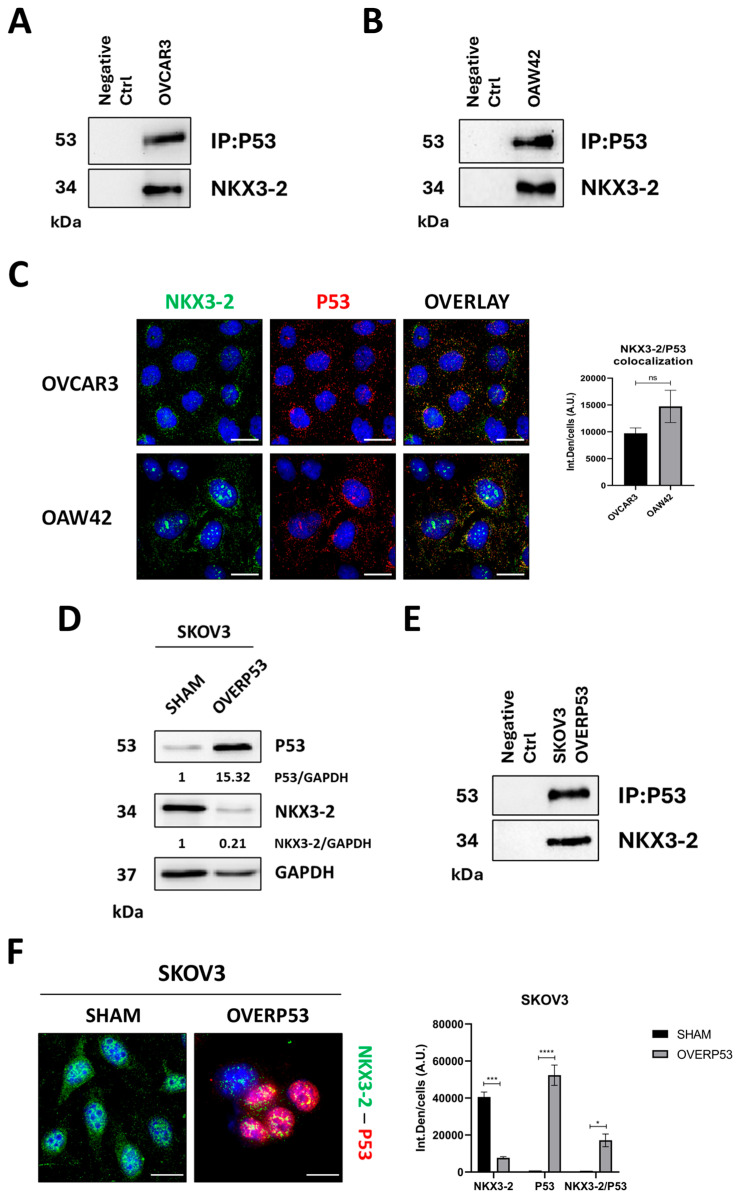
NKX3-2 interacts with p53. (**A**,**B**) Cell homogenates from OVCAR3 and OAW42 cells were processed for immunoprecipitation, and Western blotting was used to assess NKX3-2–P53 interaction. (**C**) Immunofluorescence co-staining for NKX3-2 (green)—P53 (red). Scale bar = 20 μm; magnification = 63×. The fluorescence quantification of NKX3-2–P53 co-localization is reported in the graph. (**D**) SKOV3 cells were genetically manipulated to overexpress ectopic P53. Cell homogenates were characterized by Western blotting for the expression of P53 and NKX3-2. Normalization was performed by GAPDH. Densitometric analysis is included. (**E**) Cell homogenates of SKOV3 P53-overexpressing cells were processed by immunoprecipitation, and Western blotting was used to assess the NKX3-2–P53 interaction. (**F**) SKOV3 cells genetically manipulated as described in panel D were characterized by immunofluorescence double-staining for NKX3-2 (green)—P53 (red). Scale bar = 20 μm; magnification = 63×. The quantification of NKX3-2 and P53 fluorescent signals, and NKX3-2–P53 co-localization are reported in the graph. Statistical analysis was performed using GraphPad Prism 5.0 software. Bonferroni’s multiple comparison test after *t*-test analysis (unpaired, two-tailed) was employed. Significance was considered as follows: **** *p* < 0.0001; *** *p* < 0.001; * *p* < 0.05; ns *p* > 0.05.

Next, we assessed the capability of NKX3-2 and P53 to physically interact. We performed co-immunoprecipitation in OAW42 and OVCAR3 cells (Figure 3A,B), and we detected NKX3-2 among the P53 interactors. This evidence was even confirmed by immunofluorescence (Figure 3C). To further validate this mechanism, we performed genetic manipulation in SKOV3 cells and found that the ectopic overexpression of P53 promotes a decrease in NKX3-2 levels (Figure 3D), which seems to be linked to a mechanism of interaction and sequestration of NKX3-2 by P53. Consistently, the results reported in immunoprecipitation and immunofluorescence (Figure 3E,F) confirmed the capability of P53 to interact with NKX3-2 in P53-overexpressing SKOV3 cells.

### 3.3. P53 Drives the Autophagic Degradation of NKX3-2

**Figure 4:**
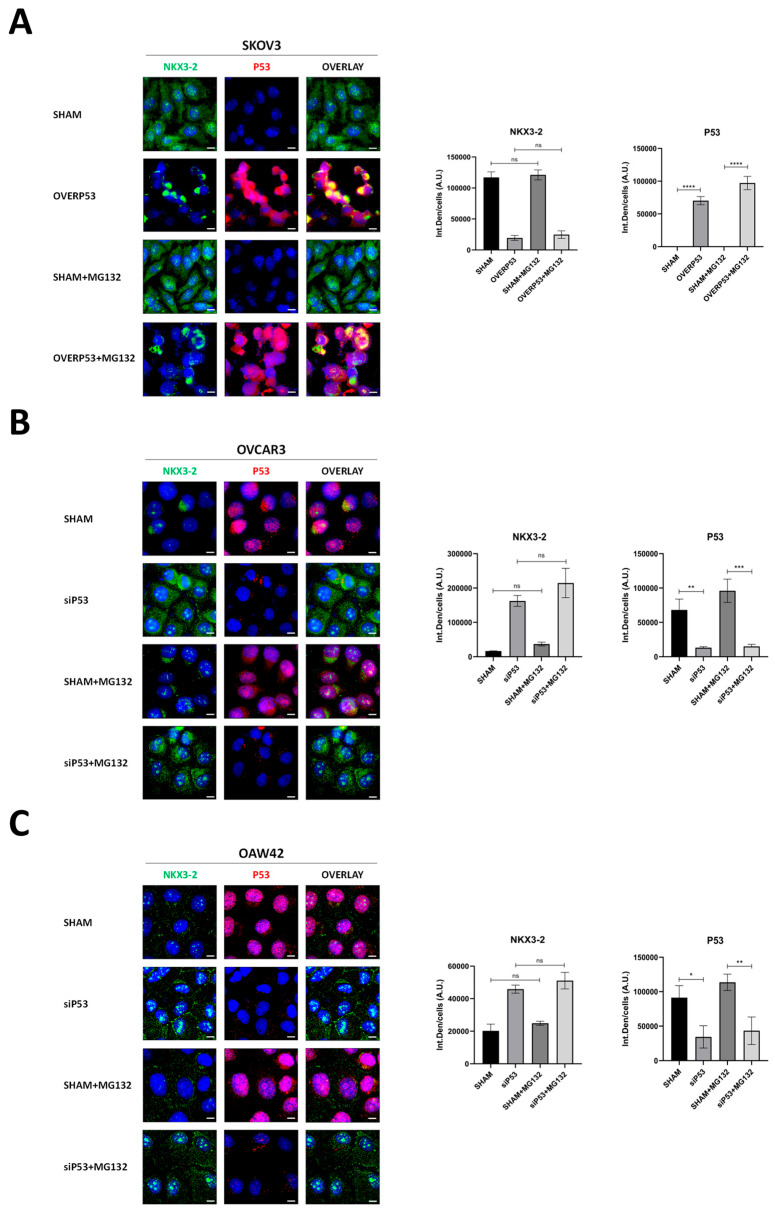
P53-induced downregulation of NKX3-2 is not mediated by proteasomal degradation. Immunofluorescence double staining for NKX3-2 (green)—P53 (red) performed on SKOV3 (**A**), OVCAR3, and OAW42 (**B**,**C**) genetically manipulated to overexpress (**A**) or silence (**B**,**C**) P53 expression. After 40 h of transfection, cells were cultured in the absence/presence of 10 µM MG132 for a further 8 h. Scale bar = 20 μm; magnification = 63×. The quantification of NKX3-2 and P53 fluorescent signals is reported in the graphs. Statistical analysis was performed using GraphPad Prism 5.0 software. Bonferroni’s multiple comparison test after One-way ANOVA analysis (unpaired, two-tailed) was employed. Significance was considered as follows: **** *p* < 0.0001; *** *p* < 0.001; ** *p* < 0.001; * *p* < 0.05; ns *p* > 0.05.

The observations that P53 interacts with and negatively modulates the expression of NKX3-2 at the post-transcriptional level led us to hypothesize that a P53-mediated degradation mechanism could be in place. To address the possible involvement of proteasomal degradation in P53-mediated NKX3-2 downregulation, we employed MG132, a potent proteasome inhibitor. The immunofluorescence double-staining shows that the addition of MG132 does not rescue the expression of NKX3-2 in the tested ovarian cell models in which P53 expression was genetically manipulated to be overexpressed (in SKOV3) or silenced (in OVCAR3 and OAW42). Indeed, no significant changes were observed in the quantification of NKX3-2 levels, as reported in the histograms (Figure 4).

**Figure 5:**
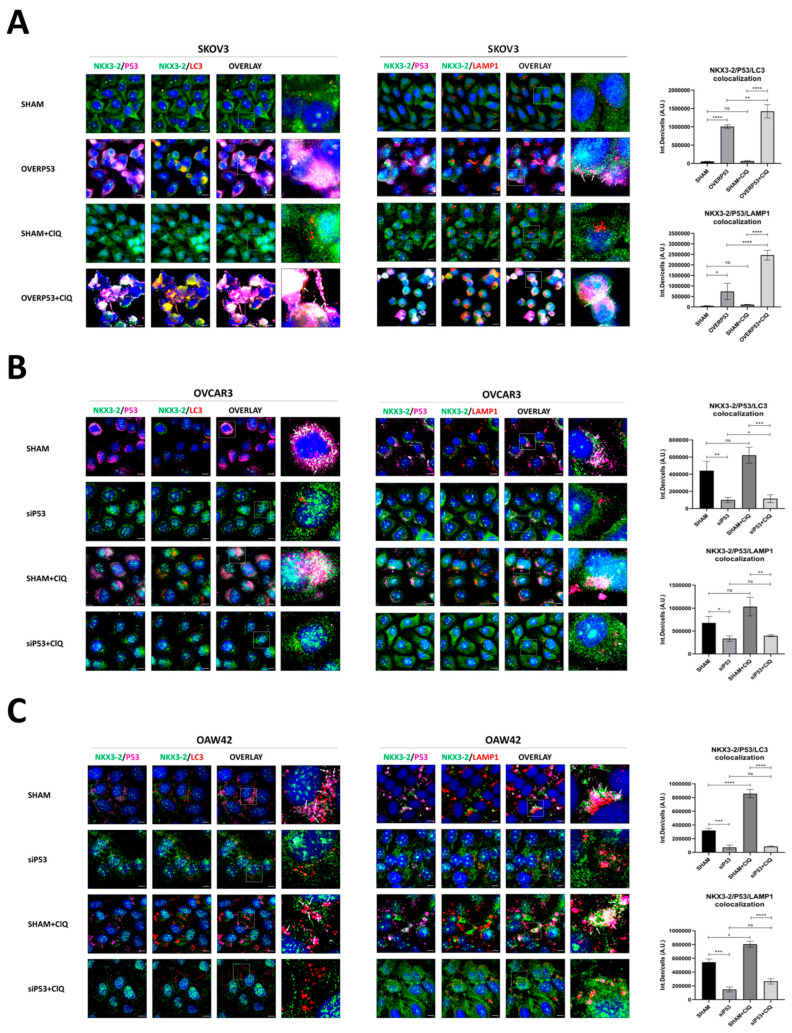
P53 induces NKX3-2 autophagic degradation. Immunofluorescence triple staining for NKX3-2 (green)—P53 (purple)—LC3 or LAMP1 (red) performed on SKOV3 (**A**), OVCAR3, and OAW42 (**B**,**C**) genetically manipulated to overexpress (**A**) or silence (**B**,**C**) P53 expression. After 40 h of transfection, cells were cultured in the absence/presence of 30 µM chloroquine (ClQ) for a further 8 h. Scale bar = 20 μm; magnification = 63×. The quantification of NKX3-2/P53/LC3 and NKX3-2/P53/LAMP1 co-localization is reported in the graphs. Statistical analysis was performed using GraphPad Prism 5.0 software. Bonferroni’s multiple comparison test after One-way ANOVA analysis (unpaired, two-tailed) was employed. Significance was considered as follows: **** *p* < 0.0001; *** *p* < 0.001; ** *p* < 0.001; * *p* < 0.05; ns *p* > 0.05.

As an alternative mechanism, we investigated whether autophagy was involved in the P53-induced post-translational downregulation of NKX3-2. We performed two immunofluorescence triple stainings for NKX3-2/P53/LC3 and NKX3-2/P53/LAMP1 on SKOV3 cells overexpressing P53, and OAW42 and OVCAR3 cells silenced for P53, respectively (Figure 5). The autophagy clearance was blocked by chloroquine (ClQ), an inhibitor of autophagosome turnover that impairs autophagosome-lysosome fusion. SKOV3 cells (that are *TP53*-null and display the highest NKX3-2 basal expression) overexpressing ectopic P53 exhibited a strong downregulation of NKX3-2, which co-localized with LC3 and LAMP1 and mainly localized in the cytoplasm. The addition of ClQ remarks on this trend (Figure 5A). On the other hand, sham-transfected OAW42 and OVCAR3 cells (which basally express low NKX3-2) displayed a strong co-localization of NKX3-2 with the autophagic marker LC3 and the lysosomal marker LAMP1, and this effect was exacerbated when cells were co-incubated with ClQ. When these cells were silenced for P53, a rescue of NKX3-2 levels in parallel to a marked reduction in its co-localization with LC3 and LAMP1 was observed (Figure 5B,C). Remarkably, these cells showed increased nuclear translocation of NKX3-2. Overall, these data suggest that P53 reduces NKX3-2 expression through protein–protein interactions, which in turn result in NKX3-2 degradation via autophagy.

**Figure 6:**
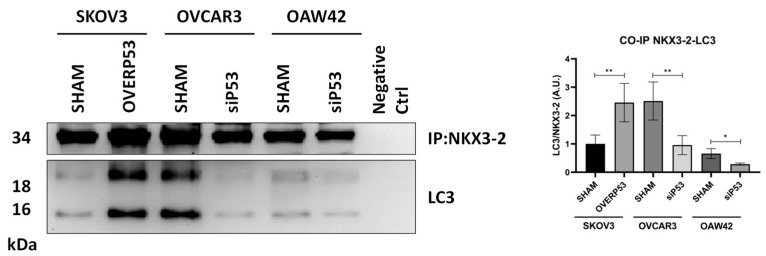
NKX3-2 interacts with LC3. SKOV3 cells were genetically manipulated to overexpress, while OVCAR3 and OAW42 cells were silenced for NKX3-2, respectively. Cell homogenates were precipitated with anti-NKX3-2 antibody, and the presence of LC3 among the interactors was assessed by Western blotting. Densitometric analysis of NKX3-2-LC3 interaction is reported in the graph. Statistical analysis was performed using GraphPad Prism 5.0 software. Bonferroni’s multiple comparison test after One-way ANOVA analysis (unpaired, two-tailed) was employed. Significance was considered as follows: ** *p* < 0.01; * *p* < 0.05.

To validate the mechanism proposed above, we performed an immunoprecipitation to assess NKX3-2/LC3 interaction (Figure 6). Consistently with the previous findings, we found that transfected SKOV3 cells overexpressing ectopic P53 displayed an increased interaction of NKX3-2 with LC3 compared to that observed in the control condition. In contrast, in OVCAR3 cells that basally show a strong interaction between the two proteins, this interaction was lost after P53 silencing. Similarly, a slight NKX3-2/LC3 interaction was also detected in OAW42 cells, which was reduced after P53 knockdown, although the trend is much less evident than in p53-silenced OVCAR3 cells.

### 3.4. Low NKX3-2 Expression and Active Autophagy Predict Prolonged Survival in Ovarian Cancer Patients

**Figure 7:**
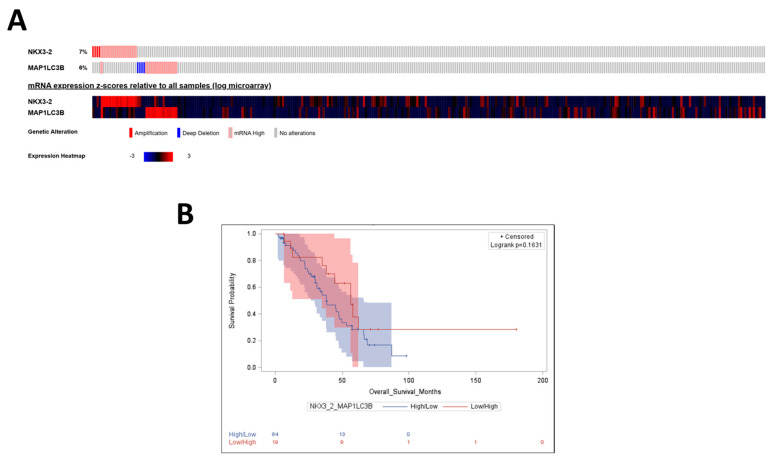
Low *NKX3-2* together with high *MAP1LC3B* expression is associated with better clinical outcomes. (**A**) The oncoprint shows the genetic alterations and mRNA expression levels of *NKX3-2* and *MAP1LC3B* in 316 ovarian cancer patients (Ovarian Serous Cystadenocarcinoma dataset, Consortium TCGA, Nature 2011). The heatmap reports the upregulated and downregulated genes in red and blue, respectively. (**B**) The Kaplan–Meier curve depicts overall survival based on *NKX3-2*/*MAP1LC3B* differential expression levels.

Finally, we assessed the prognostic significance of the NKX3-2/autophagy axis. The upper part of the oncoprint shows that only 6% of ovarian cancer patients display genetic alterations in *MAP1LC3B* (Figure 7A). In detail, four patients present a deep deletion of the *MAP1LC3B* gene, whereas sixteen exhibit an upregulation of mRNA levels. The heatmap in the bottom part shows that most of the cohort displays an opposite trend in *NKX3-2* and *MAP1LC3B* mRNA levels. Remarkably, we observed that patients bearing a tumor with low *NKX3-2* together with high *MAP1LC3B* levels (indicative of active autophagy) display a favorable clinical outcome (Figure 7B), reinforcing the view that this signature may have translational relevance for ovarian cancer.

## 4. Discussion

Ovarian cancer represents a significant challenge for modern healthcare, owing to the lack of robust biomarkers and the elevated heterogeneity of the malignancy, collectively lowering the life expectancy of patients [10]. Aiming to improve patients’ survival outcomes, the identification of novel diagnostic/prognostic predictors can provide new tools for precision and personalized medicine. There is still an urgent clinical need to identify more specific biomarkers to better refine patients’ stratification [11].

NKX3-2 is a transcriptional repressor belonging to the NK2 class of homeobox genes that plays an essential role in embryogenic development [12]. Alterations in this gene have been studied in relation to skeletal diseases [13]. A limited number of studies have reported the involvement of NKX3-2 in cancer progression [5,6,7,8]. Other studies have identified NKX3-2 as part of molecular signatures associated with metastasis or resistance/susceptibility to chemo/immunotherapy in some tumors [14,15,16,17]. However, the functional role of NKX3-2 in cancer cell biology has been poorly described so far. Recently, we reported that NKX3-2 is one of the main downstream effectors of the signaling cascade induced by LPA, promoting ovarian cancer cell motility through the downregulation of autophagy [9].

The present study aims to provide further insights into the oncogenic role of NKX3-2. Remarkably, the interrogation of TCGA clinical data reveals that high expression of *NKX3-2* was associated with unfavorable clinical outcomes in several solid tumors. Similarly, a comprehensive in silico analysis shows that NKX3-2 represents a negative prognostic indicator for liver hepatocellular carcinoma [18]. The study revealed that NKX3-2 expression positively correlates with tumor-infiltrating immune cells, collectively accelerating cancer progression by promoting immune evasion [18].

Our transcriptomic analysis performed on TCGA ovarian cancer patients’ cohort shows that *NKX3-2* expression is positively correlated with that of genes regulating apoptosis evasion, carbohydrate and lipid metabolism, and several oncogenic pathways favoring cell proliferation, motility, and macromolecule biosynthetic processes. On the other hand, *NKX3-2* expression negatively correlates with that of genes belonging to DNA damage checkpoints, P53-downstream signaling, mitochondrial oxidative metabolism, and proteolysis.

The tumor suppressor P53 acts as a central hub to integrate the response into a broad range of cellular stresses, including oncogene activation, telomere erosion, ribosomal stress, and hypoxia. Once activated, P53 regulates several intracellular processes (like cell-cycle arrest, DNA repair, apoptosis, senescence, and autophagy) and participates in the modulation of cell metabolism. Accumulating evidence demonstrated many transcription-independent roles of P53, highlighting that its functions range far beyond controlling DNA damage checkpoints. Based on these premises, P53 should be regarded not as a simple “guardian of the genome” but as a complex “guardian of the cell” [19].

We hypothesize that during clonal evolution, P53-overexpressing clones may counteract the survival of those expressing high levels of NKX3-2, while, on the other hand, in tumors lacking P53, NKX3-2-overexpressing cells may have a growth advantage and worsen the prognosis. Accordingly, multivariate survival analysis shows that ovarian cancer patients characterized by low *NKX3-2* and active P53 display better clinical outcomes, which could be related to the sensitization of cancer cells to anticancer therapy and/or P53-induced mitigation of cancer aggressiveness. Remarkably, we found that these patients exhibit a marked downregulation of a subset of genes involved in the inhibition of programmed cell death, the epithelial-to-mesenchymal transition, and pro-tumorigenic signaling pathways. In particular, we identified *SFRP2* (Secreted Frizzled Related Protein 2), *PDFGRB* (Platelet-Derived Growth Factor Receptor Beta), *IGF1/IGF2* (Insuline-like Growth Factors 1 and 2), and *ENPP1* (Ectonucleotide Pyrophosphatase/Phosphodiesterase 1). The overexpression of these markers represents an interesting signature from a prognostic point of view since their involvement in cancer cell growth, metastatic peritoneal spread, and drug resistance were previously reported in ovarian cancer [20,21,22,23,24]. Interestingly, the same group of patients (displaying low *NKX3-2*/high *TP53* expression and good prognosis) exhibits upregulation of cell cycle checkpoints, mitochondrial oxidative phosphorylation, and proteolysis. Among the transcripts belonging to these processes, we focused on *CDC5L* (Cell Division Cycle 5 Like), *OMA1* (OMA1 Zinc Metallopeptidase), and *TFAM* (Transcription Factor A, Mitochondrial). The enhanced expression of these genes represents a favorable molecular signature that predicts a chemosensitive phenotype to platinum-based therapy, given their role in the regulation of the cell cycle, the initiation of the intrinsic apoptotic cascade, and mitochondrial ROS production [25,26,27]. Starting from the evidence that P53 and NKX3-2 expression are negatively correlated (both in patients and in vitro), here we demonstrate that P53 downregulates NKX3-2 (but not vice versa), thus preventing its oncogenic activities. In detail, we show that this negative regulation is not related to the transcriptional activity of P53 nor the proteasomal degradation of NKX3-2. The experimental validation performed in different ovarian cancer cell lines proves that P53 interacts and sequesters NKX3-2 in the cytoplasm, thus promoting its degradation by the autophagy–lysosomal system. It is to be stressed that R248Q mutant P53, which affects tetramerization and DNA-binding ability, is still capable of directing NKX3-2 autophagy degradation, as occurs in OVCAR3 cells, indicating that this domain is not involved in the P53–NKX3-2 interaction. The identification of this novel mechanism is relevant from a translational point of view since the regulation of the P53/NKX3-2/autophagy axis may influence cancer cell fate and, consequently, the survival of patients.

Several studies have indicated that *TP53* mutation or loss results in limited therapy responses and worse clinical outcomes [28]. Depending on the context (e.g., mutational status, subcellular localization, and stress stimuli), P53 can promote or inhibit autophagy, a pro-survival lysosomal-driven catabolic process that is crucial for the correct turnover of aged or damaged organelles and macromolecules [29]. The deregulation of autophagy has been regarded as a key feature contributing to the development of cancer [30]. Depending on the stage of tumorigenesis, autophagy may act as a tumor-suppressive mechanism by preventing neoplastic transformation in the early steps, while supporting the survival and growth of cancer cells under stressful conditions in the advanced stages [30]. In other contexts, autophagy hyper-induction has been shown to sensitize cancer cells to chemotherapy [31,32]. Interestingly, here we show that a low *NKX3-2*/high*MAP1LC3B* signature predicts better clinical outcomes for ovarian cancer patients. In line with this finding, we have previously demonstrated that the knockdown of NKX3-2 restores autophagy and, in turn, impairs the migratory potential of ovarian cancer cells in response to a permissive microenvironment [9].

One limitation at this step is that the retrospective bioinformatic analysis was conducted on publicly available transcriptomic datasets (TCGA), which may restrict data generalizability and introduce batch effects and patient selection bias. Unfortunately, in the TCGA cohort analyzed, some information regarding age and other clinical characteristics is missing or incomplete. Additionally, there are very few patients with wild-type *TP53* in TCGA dataset, thus limiting the comparison with *TP53*-mutated patients. Further investigations are needed to extend the translational relevance of our study, possibly with a larger number of cases for each genetic group. In line with this view, we are about to start a pilot study establishing our patients’ cohort in which we will address the possible correlations between the P53/NKX3-2/autophagy axis and tumor staging, grading, and therapeutic outcomes. Additionally, an examination of the peritoneum and/or fallopian tube and a comparison of NKX3-2 expression in non-cancer patients (e.g., controls, subjects that undergo surgery for hysterectomy) and cancer patients are instrumental to strengthen the translational relevance of our findings.

## 5. Conclusions

In conclusion, our data suggest that NKX3-2 represents a negative prognostic marker and may provide the basis for the identification of a molecular signature capable of predicting the clinical outcome of ovarian cancer patients, a crucial aspect for the development of a more effective therapeutic approach.

## Data Availability

All data and materials are published in the manuscript; Supplementary Materials are published on the journal website.

## Supplementary Materials

The following supporting information can be downloaded at: https://www.mdpi.com/article/10.3390/cells14110765/s1, Supplementary Figure S1: *NKX3*-2 expression is associated with poor prognosis in different solid tumors. Patients bearing different cancers, like ovarian cancer (A; *p* = 0.0341), glioma (B; *p* < 0.001), colorectal cancer (C; *p* = 0.0016), kidney carcinoma (D; *p* = 0.0023), liver cancer (E; *p* = 0.0027), and breast cancer (F; *p* = 0.0382), with high *NKX3*-2 mRNA expression correlate with a poor prognosis as shown by the Kaplan–Meier plots obtained from the interrogation of the TGCA datasets; Supplementary Figure S2: *NKX3-2* positively correlates with pro-tumorigenic metabolism and negatively correlates with p53-related pathways. Expression data were retrieved from TCGA ovarian cystadenocarcinoma dataset, and patients were stratified based on *NKX3-2* mRNA expression (high vs. low). Graphs report the number of transcripts belonging to each biological process and pathway positively (A) or negatively (B) associated with *NKX3-2* mRNA expression; Supplementary Figure S3: P53-mediated downregulation of NKX3-2 does not change the cytoplasmic/nuclear translocation of NKX3-2. The subcellular localization of NKX3-2 was monitored by differential cytoplasmic/nuclear protein extraction by using the NE-PER kit. The graph reports the quantification of the cytoplasmic/nuclear ratios in cells genetically manipulated to overexpress (SKOV3) or knockdown (OVCAR3 and OAW42) P53 expression. Statistical analysis was performed using GraphPad Prism 5.0 software. Bonferroni’s multiple comparison test after One-way ANOVA analysis (unpaired, two-tailed) was employed. Significance was considered as follows: ns *p* > 0.05; Supplementary Figure S4: P53 accumulation by Nutlin-3 downregulates NKX3-2. OAW42 cells were treated with 5 µM Nutlin-3 for 48 h and then characterized for Western blotting (A) and immunofluorescence double-staining (B) to monitor the expression and subcellular localization of P53 and NKX3-2. The densitometric analysis of Western blotting is reported.
