# Supplementary material for: P53-Induced Autophagy Degradation of NKX3-2 Improves Ovarian Cancer Prognosis
Source: Cells. 2025 May 22;14(11):765. doi: 10.3390/cells14110765 (PMC12153924; doi:10.3390/cells14110765)
Supplement: Supplementary file 1 [file cells-14-00765-s001.zip › cells-3638994-supplementary.pdf]

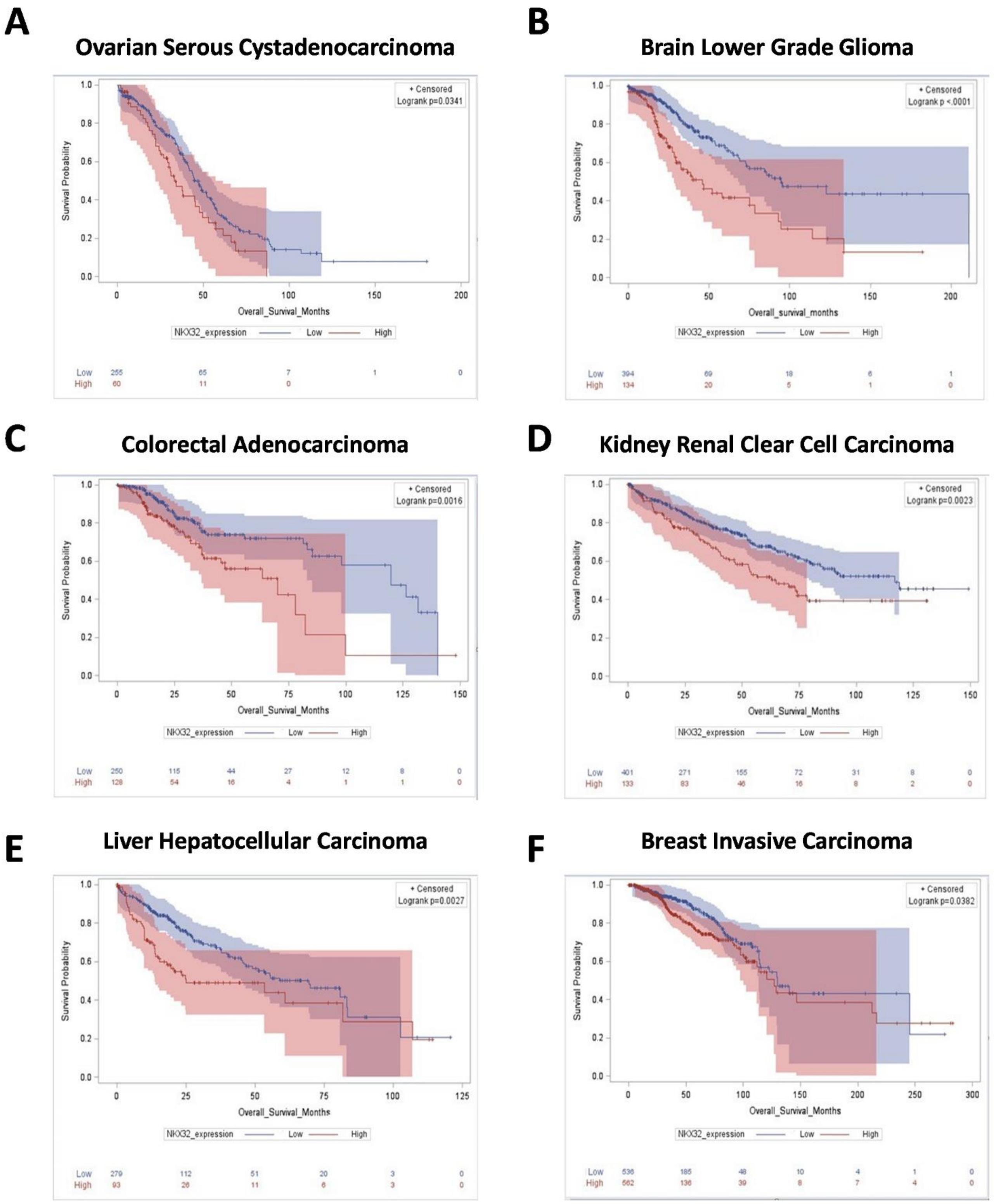

Supplementary Figure S1. ***NKX3-2* expression is associated with poor prognosis in different solid tumors.** Patients bearing different cancers, like ovarian cancer (A; p = 0.0341), glioma (B; p < 0.001), colorectal cancer (C; p = 0.0016), kidney carcinoma (D; p = 0.0023), liver cancer (E; p = 0.0027), and breast cancer (F; p = 0.0382), with high *NKX3*-2 mRNA expression correlate with a poor prognosis as shown by the Kaplan-Meier plots obtained from the interrogation of the TGCA datasets.

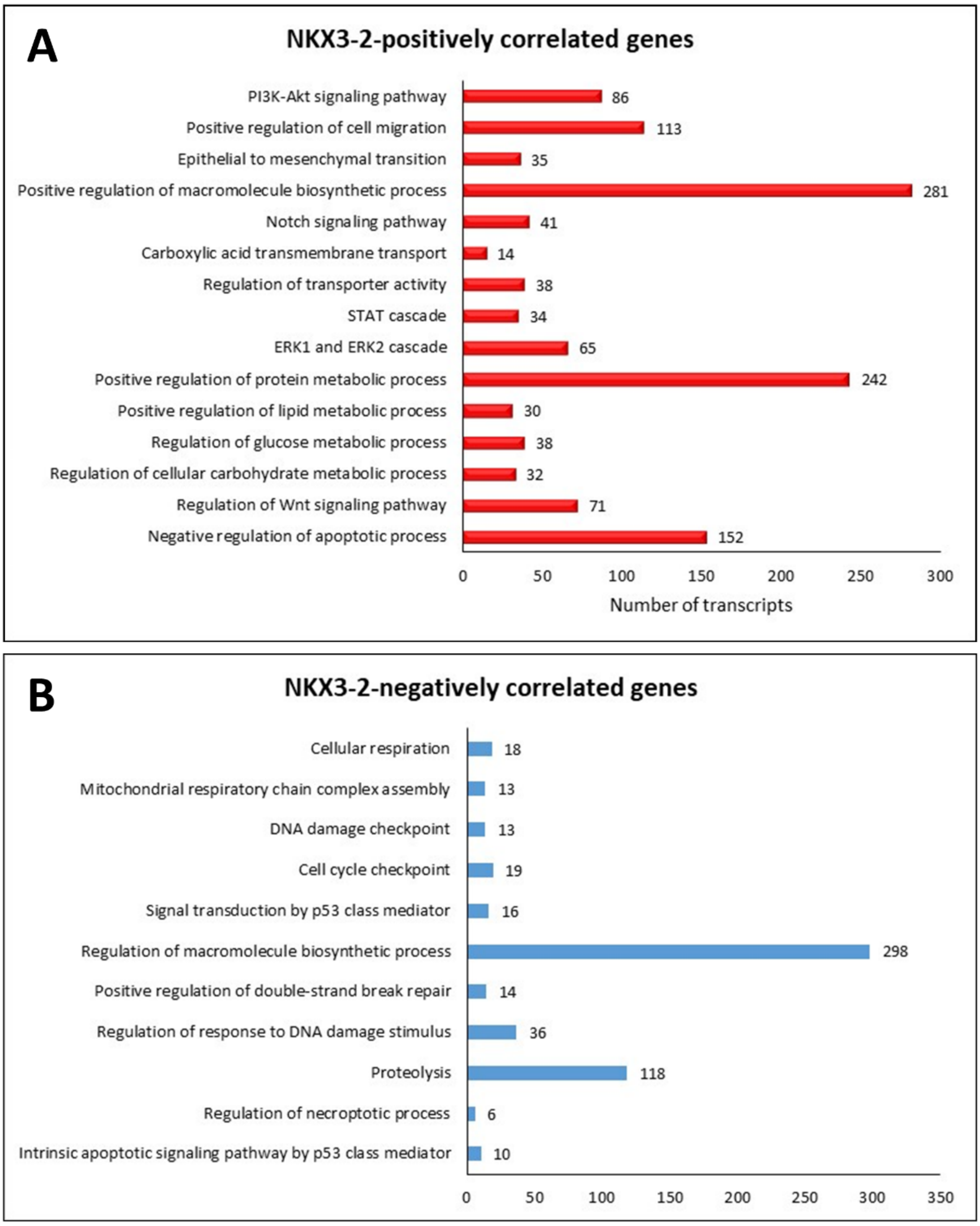

Supplementary Figure S2. *NKX3-2* positively correlates with pro-tumorigenic metabolism while negatively correlates with P53-related pathways. Expression data were retrieved from TCGA ovarian cystadenocarcinoma dataset, and patients were stratified based on *NKX3-2* mRNA expression (high vs. low). Graphs report the number of transcripts belonging to each biological process and pathway positively (A) or negatively (B) associated with *NKX3-2* mRNA expression.

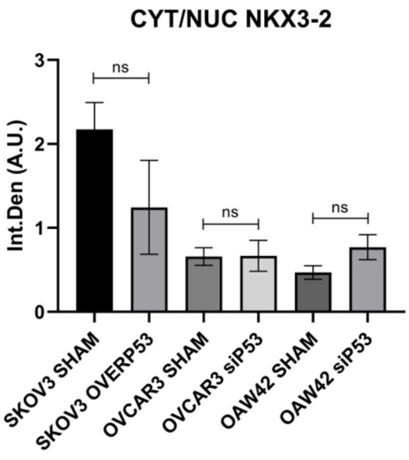

Supplementary Figure S3. P53-mediated downregulation of NKX3-2 does not change the cytoplasmic/nuclear translocation of NKX3-2. The subcellular localization of NKX3-2 was monitored by differential cytoplasmic/nuclear protein extraction by using NE-PER kit. The graph reports the quantification of the cytoplasmic/nuclear ratios in cells genetically manipulated to overexpress (SKOV3) or to knockdown (OVCAR3 and OAW42) P53 expression. Statistical analysis was performed using GraphPad Prism 5.0 software. Bonferroni's multiple comparison test after One-way ANOVA analysis (unpaired, two-tailed) was employed. Significance was considered as follows: ns $p > 0.05$.

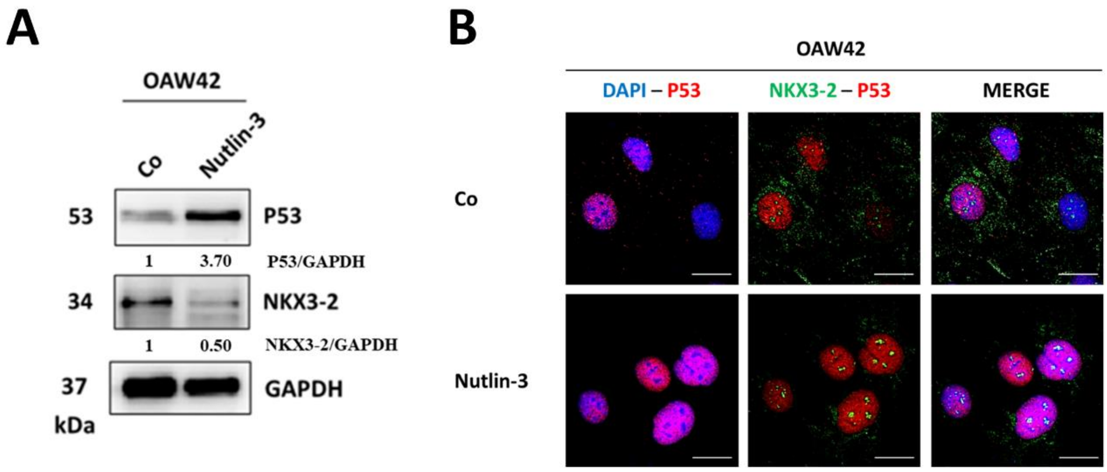

Supplementary Figure S4. P53 accumulation by Nutlin-3 downregulates NKX3-2. OAW42 cells were treated with 5 μM Nutlin-3 for 48 hours and then characterized for Western blotting (A) and immunofluorescence double-staining (B) for monitoring the expression and subcellular localization of P53 and NKX3-2. The densitometric analysis of Western blotting is reported.
